# Conditioning of the Cortisol Awakening Response in Healthy Men: Study Protocol for a Randomized Controlled Trial

**DOI:** 10.2196/38087

**Published:** 2023-06-19

**Authors:** Fabian Wolters, Henriët van Middendorp, Omer Van den Bergh, Nienke R Biermasz, Onno C Meijer, Andrea WM Evers

**Affiliations:** 1 Health, Medical and Neuropsychology Unit Faculty of Social Sciences Leiden University Leiden Netherlands; 2 Leiden Institute for Brain and Cognition Leiden University Leiden Netherlands; 3 Health Psychology University of Leuven Leuven Belgium; 4 Division of Endocrinology Department of Medicine Leiden University Medical Center Leiden Netherlands; 5 Medical Delta Leiden University, Technical University Delft and Erasmus University Rotterdam Leiden, Delft, Rotterdam Netherlands; 6 Department of Psychiatry Leiden University Medical Center Leiden Netherlands

**Keywords:** conditioning, cortisol, cortisol awakening response, sleep, olfactory learning

## Abstract

**Background:**

The hormone cortisol plays important roles in human circadian and stress physiology and is an interesting target for interventions. Cortisol varies not only in response to stress but also as part of a diurnal rhythm. It shows a particularly sharp increase immediately after awakening, the cortisol awakening response (CAR). Cortisol can be affected by medication, but it is less clear whether it can also be affected by learning. Animal studies have consistently shown that cortisol can be affected by pharmacological conditioning, but the results in humans have been mixed. Other studies have suggested that conditioning is also possible during sleep and that the diurnal rhythm can be conditioned, but these findings have not yet been applied to cortisol conditioning.

**Objective:**

The objective of our study was to introduce a novel avenue for conditioning cortisol: by using the CAR as an unconditioned response and using scent conditioning while the participant is asleep. This study investigates an innovative way to study the effects of conditioning on cortisol and the diurnal rhythm, using a variety of devices and measures to make measurement possible at a distance and at unusual moments.

**Methods:**

The study protocol takes 2 weeks and is performed from the participant’s home. Measures in week 1 are taken to reflect the CAR and waking under baseline conditions. For the first 3 nights of week 2, participants are exposed to a scent from 30 minutes before awakening until their normal time of awakening to allow the scent to become associated with the CAR. On the final night, participants are forced to wake 4 hours earlier, when cortisol levels are normally low, and either the same (conditioned group) or a different (control group) scent is presented half an hour before this new time. This allows us to test whether cortisol levels are higher after the same scent is presented. The primary outcome is the CAR, assessed by saliva cortisol levels, 0, 15, 30, and 45 minutes after awakening. The secondary outcomes are heart rate variability, actigraphy measures taken during sleep, and self-reported mood after awakening. To perform manipulations and measurements, this study uses wearable devices, 2 smartphone apps, web-based questionnaires, and a programmed scent device.

**Results:**

We completed data collection as of December 24, 2021.

**Conclusions:**

This study can provide new insights into learning effects on cortisol and the diurnal rhythm. If the procedure does affect the CAR and associated measures, it also has potential clinical implications in the treatment of sleep and stress disorders.

**Trial Registration:**

Netherlands Trial Register NL58792.058.16; https://trialsearch.who.int/Trial2.aspx?TrialID=NL7791

**International Registered Report Identifier (IRRID):**

DERR1-10.2196/38087

## Introduction

### Background

**Figure 1:**
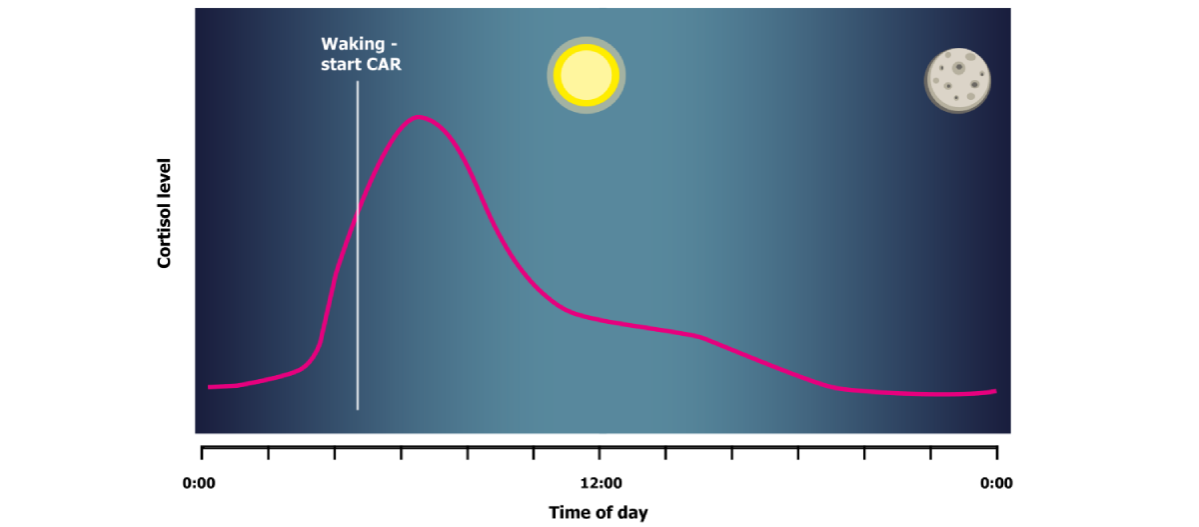
Stylized representation of a typical change in cortisol levels over a 24-hour period. CAR: Cortisol Awakening Response.

The hormone cortisol has far-reaching effects throughout the body, mainly influencing metabolism but also being involved in systems as varied as the immune system [1], memory [2,3], and the sleep-wake cycle [4]. Cortisol is secreted from the adrenal gland as the end product of the hypothalamic-pituitary-adrenal (HPA) axis, a system of cascading secretions that originate in the hypothalamus. Cortisol is perhaps best known for its role in the stress response [5], and thus is often called the “stress hormone” in everyday parlance. However, cortisol does not just fluctuate in response to stress; it also follows several rhythms, including a diurnal rhythm. Cortisol levels are highest in the early morning and decline throughout the day and evening, with levels increasing again in the second half of the night [6]. A visual representation of a typical rhythm is shown in Figure 1. The onset of pulsatile secretion varies, but a strong peak is seen in cortisol increase in the period immediately after awakening in most participants [7]. This peak is referred to as the cortisol awakening response (CAR). The CAR has been extensively studied and has been shown to be generally consistent in size within an individual [7,8] but also influenced by situational variables [9], such as sleep duration in the preceding night [10]. Befitting the role of the stress hormone, the most reliable of the situational variables are stress-related [11]. Reviews of relevant studies suggest that the CAR is partially responsible for preparing the body in anticipation of the events and challenges of the day ahead [9,10]. Anticipation of a stressful and challenging day may lead to an increased CAR [12], while an intervention that reduces stress showed a corresponding decrease in the CAR [13]. Meanwhile, patients with severe amnesia, presumably not expecting anything from the coming day, seem to show no CAR at all [14].

Apart from stress and diurnal rhythms, cortisol levels can also be influenced more directly by medical interventions, whether intentionally or as a side effect. For example, sumatriptan is a serotonin receptor agonist taken to help with migraines, but it also reduces serum levels of adrenocorticotropin, and will reduce cortisol levels as a result [15]. Corticosteroids, such as dexamethasone, are a synthetic form of natural cortisol and can be used as cortisol replacement or to treat inflammation. Because cortisol is one part of an interconnected system, these types of medication can have indirect effects. Increased serum cortisol levels will normally trigger negative feedback to the hypothalamus and pituitary, the first links in the HPA axis, which leads to serum cortisol levels decreasing [16]. Corticosteroids trigger the same mechanism, which means that taking them lowers cortisol levels in the long term. Thus, it is always important to consider the timeframe and downstream effects when estimating the effect of any form of medication on cortisol levels.

Once the body is familiar with the effects of cortisol-affecting medication, learning processes can modulate their effects. These generally arise through the process of classical conditioning. Classical conditioning occurs when a natural reaction, the unconditioned response, which is normally caused by a stimulus termed the unconditioned stimulus (UCS), is repeatedly paired with a different, traditionally neutral stimulus. Once an association is established, the paired stimulus, known as the conditioned stimulus (CS), may evoke a response even when the UCS is not present; in this case, the response is known as the conditioned response [17]. These principles can be applied to cortisol-affecting medication. After repeated pairings, the CS will “remind” the body of the UCS and evoke either the cortisol change produced by the medication or a compensatory feedback mechanism [18,19]. A review of human and animal studies [20] found that this type of pharmacological conditioning was effective in animals, with 13 out of 15 studies finding the expected effect but less often tested and not as effective in humans. Of the human studies performed on this topic, 2 used medication that indirectly affects cortisol levels as a UCS: Benedetti et al [21] used sumatriptan, which reduces cortisol levels, while Petrakova et al [22] used corticotropin-releasing hormone, an upstream factor in the HPA axis. In the study by Benedetti et al [21], participants were injected with sumatriptan on 2 consecutive days, and on the third day, they received a saline injection presented as a 50/50 chance of sumatriptan or saline. Cortisol levels on the third day decreased in response to the injection in this group, while no effect was observed in the group that received the same injection without the previous 2 days of conditioning. Petrakova et al [22] used 2 conditioning trials 3 days apart, where participants received a distinctly flavored and colored drink combined with an injection of corticotropin-releasing hormone. After another 3 days, the participants were tested with a saline (placebo) injection on 2 subsequent days. Compared with a control group that also received saline injections in the conditioning phase, the participants showed an increase in cortisol during conditioning, but not in response to saline.

Two other studies relied on a direct increase in cortisol. Sabbioni et al [23] used dexamethasone, a glucocorticoid, while Tekampe et al [19] used hydrocortisone. In both cases, the CS was a strongly flavored drink combined with oral intake of the UCS on 3 occasions during the conditioning period. The results here are mixed again. Both studies indicate that cortisol increased after conditioning, but the results are inconclusive because of low power or group differences at baseline, respectively. On the basis of the 4 studies investigating this topic, it remains unclear to what extent and in what situations cortisol levels are subject to classical conditioning in humans. It may be worthwhile to test cortisol conditioning in other situations.

Association learning is generally performed while the learner is fully conscious, but a line of studies suggests that it is also possible during sleep. A series of studies by the same research group [24-26] have shown that associations between a scent and another stimulus can be formed during sleep. While these stimuli are relatively subtle, they can lead to relevant behavioral effects; 1 study found that coupling the scent of smoke with an aversive stimulus for one night led to a modest reduction in smoking for several days afterward [25]. Odors seem to be particularly suited for this type of learning, because they do not wake sleeping individuals but seem to reduce arousal and wakefulness [27] and seem to function as a strong memory stimulus in general [28]. This idea is supported by other exploratory studies suggesting that presenting a scent during sleep can reactivate memories. For example, 1 study had participants perform a problem-solving task in the evening and simultaneously present a scent. On the subsequent night, some participants were reexposed to the scent, and these participants imagined and applied more creative solutions to the problem the next day compared with participants who received a different or no scent [29]. The authors suggest that this effect was the result of reactivating memory of the task during sleep. Another study [30] similarly presented a scent during sleep that was also present during a task performed the evening before, in this case using a visual memory task. In addition, while the scent was presented, functional magnetic resonance imaging measures were taken to examine brain activity. The results showed that the presentation of the scent activated the hippocampus, known for its role in declarative memory, and improved memory performance the following day, suggesting that scents presented during sleep can reactivate memories and influence objective outcomes.

Two animal studies have indicated that this type of learning may apply not only to associations relevant outside of sleep but also to the workings of the diurnal rhythm itself. In 1 study, a CS that was previously associated with light exposure was found to be able to trigger behavioral awakening patterns and awakening-related activity in the suprachiasmatic nucleus [31]. In another study, a stimulus linked to light deprivation was able to trigger increased melatonin levels related to the start of sleep [32]. To our knowledge, these results have not been replicated in humans. However, 1 publication suggests that the diurnal pattern of cortisol concentration can be influenced by learning [33]. When participants were informed that they would be awoken 3 hours before normal, they showed an anticipatory increase in adrenocorticotropin several hours before awakening. This response was not present when the participants were not given any warning, regardless of whether they were awoken 3 hours before normal or simply allowed to keep sleeping. This suggests that the human diurnal rhythm may be subject to learning effects just as it is in animals.

### Objective

This study builds on these separate lines of evidence by investigating whether the CAR can be conditioned during sleep and moved to an earlier point in the night. Manipulation of the CAR could help evoke the benefits associated with a high CAR, such as a more alert and prepared awakening on busy days, or a low CAR, such as a more restful awakening. The association between cortisol and the diurnal cycle may indicate that such effects could be helpful in normalizing the sleep-wake cycle when it has been disturbed, as seen in conditions such as seasonal affective disorder [34] or delayed sleep phase disorder [35]. These effects are especially valuable considering that they could be evoked without the use of medication and the associated side effects.

Owing to the interindividual reliability and strength of the CAR, it has the potential to be an effective unconditioned response. By exposing participants to a distinctive odor in the half hour before awakening, we expect that an association can be created between the cortisol increase in this period and the distinctive scent. According to the principles of classical conditioning, the scent alone should also be able to evoke an increase in the CAR and potentially accompanying feelings of wakefulness, even when administered hours before the regular time of awakening.

## Methods

### Ethics Approval and Dissemination

The study was approved by the Medical Ethics Committee of the Leiden University Medical Centre, Leiden, the Netherlands (case NL62812.058.017), who will be notified of major changes. The study is performed in accordance with the principles of the Declaration of Helsinki and Dutch Medical Research Involving Human Subjects Act (WMO). The study was preregistered at the Dutch trial register [36]. The results will be disseminated in peer-reviewed journals, international conferences, and the PhD thesis of the first author.

### Participants

Participants are recruited from the local Dutch population and compensated with €100 (US $110.25) for participation. Flyers displaying a summary of the study inclusion criteria and procedures as well as the compensation amount are disseminated on social media through word of mouth by students and teachers, in university buildings, and in local student housing. Owing to the novel and exploratory nature of the study design, no sufficiently similar studies are available to form the basis of a power analysis. Studies on similar topics have used different outcome measures and statistical analyses and thus do not offer a usable comparison. Therefore, it was decided to run a pilot study and include as many participants as possible within the grant-permitted timeframe, which resulted in 50 inclusions and 42 participants completing the full protocol. It should be noted that the most similar studies available use a comparable number of participants [24,25,37].

To avoid confounding in the measurement of the CAR by menstrual cycle or age [38], only male individuals between the ages of 21 and 34 years are included, and participants are excluded if their answers during screening indicate obesity (defined as BMI >30 kg/m^2^), brain damage, or HPA axis–related endocrine disorders (Cushing disease or Addison disease). Participants currently taking medication (including glucocorticoid medication) or who have a habit of heavy drinking or recreational drug use are also excluded. Participants are screened for severe somatic or psychiatric conditions that would interfere with the study protocol using the Mini-International Neuropsychiatric Interview Plus (MINI-Plus) [39]. To ensure a regular sleep pattern, participants are screened using the Pittsburgh Sleep Quality Index (PSQI) [40] and excluded if they experience jetlag or participate in shift work up to 1 week before participation. To ensure that participants are able to properly detect the scents used in the study, they are excluded if they indicate that they have olfactory impairments. Participants are also excluded if they report allergies or sensitivity to perfume or similar substances that might indicate an adverse reaction to the scents used in the study or if they show an allergic reaction to the scents in the course of the study.

Ongoing health and stress are checked at several points from 1 week before participation up to the conclusion of the study, and participants are encouraged to report any changes between these points. Participation is stopped if a participant uses glucocorticoid medication, has high levels of stress, or falls ill during the study period. The participants are asked to refrain from alcohol and drug use for the duration of the study. Caffeine use is permitted for consistency with existing habits but is monitored. They note their regular awakening time and are requested to set their alarm at exactly the noted time and maintain a steady sleep rhythm for the duration of the study. All participants sign an informed consent before participation.

Upon inclusion, participants are randomized to the experimental or control group through a pregenerated random number list, set to randomize in blocks of 4. Owing to procedural constraints (see the *Procedure* section), the study is single-blinded. Participants are not informed about the existence of a control group or the purpose of the study until the debriefing at the end of the study; instead, they are told that the study examines the effect of certain scents on sleep.

### Design

**Figure 2:**
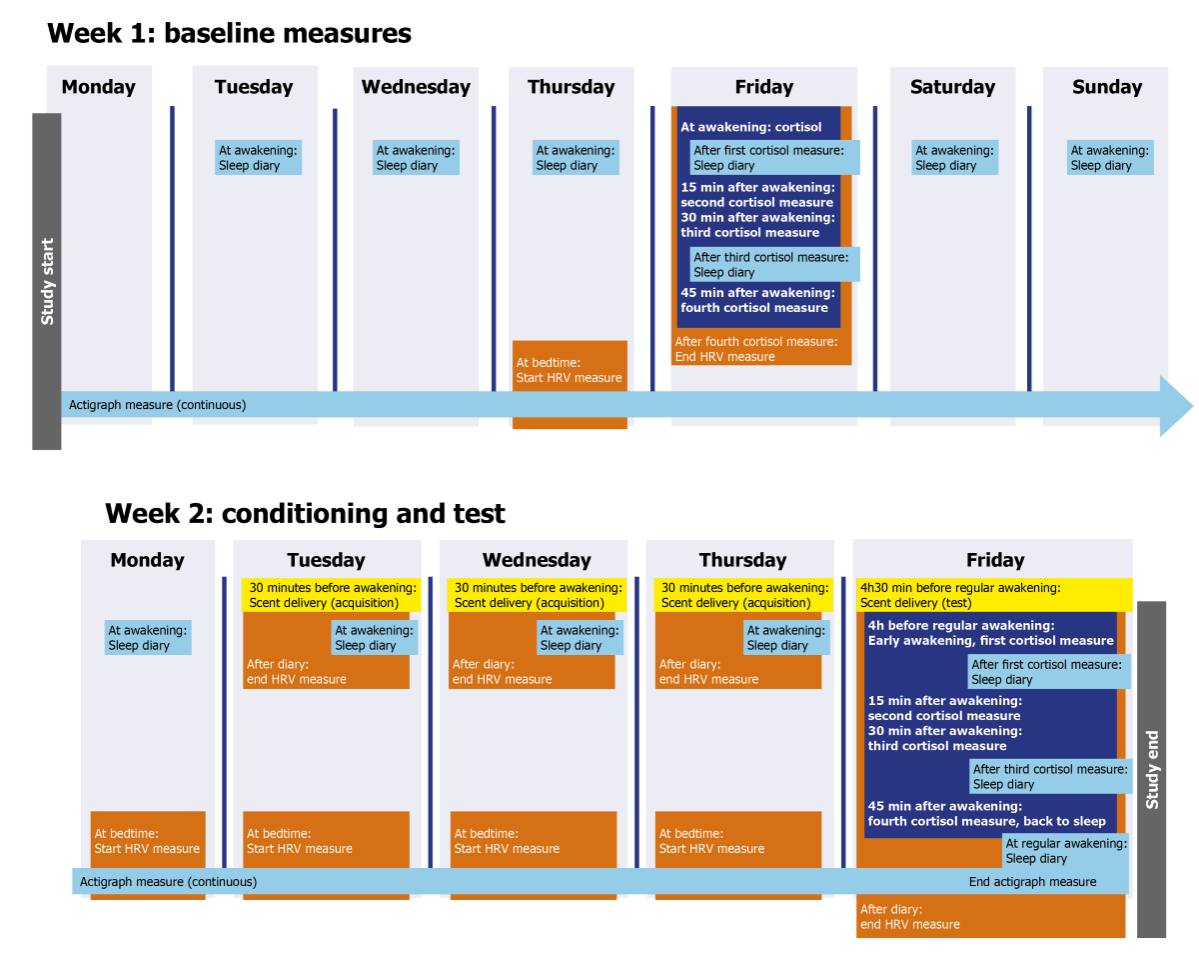
Study design of the protocol conditioning the cortisol awakening response. See Design and Procedure section for details on the content of each measurement. HRV: heart rate variability.

The study design is summarized in Figure 2. Participants follow a 2-week protocol, with all measurements taking place in their own homes. The first week functions as a baseline measure. In the second week, the scent conditioning takes place on Tuesday through Thursday morning. Participants are exposed to a scent half an hour before awakening, with the aim of conditioning the scent to the CAR. The 30-minute interval was chosen to match the CAR in length and to increase the chance that the participant is still asleep for the length of the scent presentation, while still clearly preceding the CAR and minimizing the time between CS and CAR. It is presupposed that the CAR naturally functions as an unconditioned response to daylight and other zeitgebers that set the diurnal rhythm that serves as the UCS. The final night of the study functions as the test phase. To test whether conditioning has taken place, participants in the experimental group are asked to set their alarm 4 hours earlier than normal, and the scent device releases the trained scent half an hour before this time. This way, the CS is present, while the UCS is not. Participants in the control group wake up at the same time, but for them, the scent has surreptitiously been replaced the previous day with a different, novel scent, so that neither the CS nor the UCS is present. We expect that in the experimental group, the scent (CS) will evoke the CAR and lead to an increase in cortisol levels. While the process of awakening may lead to an increase in cortisol levels in both groups, the presence of the CS would cause this increase to be more pronounced in the experimental group.

### Measurements

#### Scent Delivery System and Scents Used

Scents are delivered using the Air/Q-100 and Air/Q-160 devices and accompanying scent cartridges (Prolitec Inc). The scents used in the study were “Blue Wood” (Prolitec reference 9083) and “Marsala” (Prolitec reference 9141), with the latter used as the control scent during the last night for the participants in the control group and the former for all other instances. The scents were previously piloted to ensure that they were sufficiently distinctive. The devices release the scent at intervals of approximately 2 minutes every 9 minutes. The exact length of the delivery was dependent on the settings of the device, which were adjusted to the size of the participant’s bedroom based on the manufacturer’s suggestions. Custom-made containers were used to muffle the noise generated by the scent delivery devices.

#### Physiological Measures

##### Cortisol Awakening Response

Participants take saliva measures themselves on 2 consecutive Fridays of the experiment to measure cortisol levels. A total of 4 samples are taken each week: immediately after awakening, 15 minutes after awakening, 30 minutes after awakening, and 45 minutes after awakening. Participants are instructed not to eat, brush their teeth, smoke, exercise, shower, or drink anything other than water between awakening and taking the final sample to avoid any contamination of the samples or confounding of cortisol values [38]. Saliva is collected using sealed swabs designed for cortisol collection (Salivettes; Sarstedt AG and Co). Cortisol is assessed in saliva with a competitive electrochemiluminescence immunoassay using a Modular Analytics E602 immunoanalyzer (Roche Diagnostics). Cortisol activities are measured and expressed in nmol/L. Participants track the times when they take the saliva measures in a smartphone app, using a phone that they receive from an experimenter and use throughout the study to allow the researchers to verify that they were awake and took the required samples at the correct time. Because cortisol samples remain stable at varying temperatures for up to 5 days [41], participants were not required to cool or freeze the samples once collected. Samples were retrieved by a researcher and frozen on the same day they were collected.

The cortisol measure requires participants to be awake, but the scent conditioning procedure operates for half an hour before the participant wakes. To investigate whether the conditioning procedure has an effect before awakening, heart rate variability and actigraphy measures are included that operate throughout the night. These measures also allow for an additional check for whether the participant did indeed awake at the scheduled time.

##### Heart Rate Variability

Heart rate is measured using Polar H7 and H10 heart rate sensors (Polar Electro Oy) connected to the Heart Rate Variability Logger smartphone app [42] using the same phone as the one required for registering cortisol measures. Participants equip themselves with the heart rate monitor before going to bed on the first Thursday and every day of the second week, so that it stays on throughout the night. On days when they perform the saliva measures, the monitor is kept on until the end of those measures (ie, 45 minutes after awakening) so that any changes in heart rate that accompany the change in cortisol levels during this period can also be measured.

##### Actigraphy

Actigraphy is performed using Motionwatch 8 wearable motion detectors (CamNtech Ltd). Measures are taken over the entire 2-week period with 1-minute epochs.

#### Questionnaires

All questionnaires used have previously been found to have adequate validity and reliability.

For screening, the participants complete the PSQI [40]. Scores of 5 or higher indicate poor sleep and are used as an exclusion criterion. In addition, participants are screened for mental disorders using the MINI-Plus [39,43] and excluded if they meet the criteria for any disorder.

Several personality characteristics are measured upon inclusion, to check for possible interaction with the main outcome. Specifically, the questionnaires used are the extraversion and neuroticism scales of the Eysenck Personality Questionnaire [44,45] to measure extraversion and neuroticism, the Depression Anxiety Stress Scale-21 (DASS-21 [46]) to measure symptoms of depression and anxiety, the Life Orientation Test–Revised [47] to measure optimism, the Perceived Stress Scale [48] to measure general appraisal of stress, and the Morningness-Eveningness Questionnaire [49] to measure chronotype.

Every day of the study, immediately after awakening or after the first saliva measure, participants complete a web-based diary on their phone or PC, which includes several questionnaires. To measure positive and negative affect, the Positive and Negative Affect Schedule (PANAS [50,51]) is used. Stress is measured using a custom-made Numerical Rating Scale (NRS) asking participants to score themselves between 0 (“not at all”) and 100 (“very much”) on 7 stress-related words [19,52]. Vitality is measured using the Subjective Vitality Scale (SVS [53]), and fatigue using the fatigue subscale of the Checklist Individual Strength (CIS [54]) adjusted to measure the current state of fatigue.

The sleep diary also includes custom-made daily questions about habits that indicate or may influence sleep quality and patterns. Participants are asked about their meal times; their intake of caffeine, alcohol, tobacco, medication, and recreational drugs; sporting activities; the time at which they fell asleep the previous night and the time at which they awoke in the morning; any nighttime awakenings; and their perceived sleep quality.

### Procedure

An experimenter screens interested applicants through a telephone interview consisting of questions about relevant exclusion criteria (*Participants* section) and vocal administration of the PSQI (no data are saved). If participants meet the criteria, a follow-up screening session at Leiden University is scheduled to obtain written informed consent, perform a more elaborate check of mental and physical health, and to further explain the procedure and schedule meetings. This screening session consists of several questions about sleep, stress, and health and the Mini-International Neuropsychiatric Interview (MINI) plus international psychiatric interview. Participants who pass the screening also complete the Eysenck Personality Questionnaire – Revised Short Scale, Life Orientation Test–Revised, DASS-21, Perceived Stress Scale, and Morningness-Eveningness Questionnaire in this session. To ensure that the participants know how to take a sample of their saliva, they practice using the swabs used in the study under the supervision of a member of the research team.

The procedure of the actual experiment takes place over a period of 2 weeks, always starting on a Monday and ending on the second Friday. All measurements are performed at the participants’ homes; an experimenter visits the participants at several points to deliver and take equipment, check on participants’ health and stress, offer instructions on study procedures, and check whether all measurements have been completed successfully. Participants indicate their regular time of awakening before the experiment starts and instructed to set their alarm for this time for every day of the study. They are instructed to try to fall back asleep if they wake earlier than the indicated time. However, if they are fully awake, they should start all measurements at the earlier time of awakening.

The measures begin once an experimenter delivers the equipment on Monday of the first week. Participants are checked for exclusion criteria, instructed in the procedure for the coming days, and put on the actograph watch. The (waterproof) actograph watch will be worn continuously until the study ends, barring special circumstances such as high-contact sports or interactions with sensitive equipment where the watch could cause an issue, in which case it can be removed for the duration of the activity. Up until the evening of Thursday of week 1, participants fill in the web-based diary each morning at awakening and indicate their wake and sleep times on the actograph. Thursday evening, they put the heart rate monitor around their chest and wear it for the following night. On Friday morning, when waking up at their regular time of awakening, the first saliva measure is taken. Participants then complete the morning’s sleep diary, followed by another saliva measure 15, 30, and 45 minutes after awakening. An additional diary, where the PANAS, Stress NRS, SVS, and CIS-fatigue are completed again, is taken after the third saliva measure. After the last saliva measure, the participants remove the heart rate monitor. An experimenter arrives later in the day to take saliva measures for analysis.

During the weekend and on the morning of the following Monday, participants only complete the web-based sleep diary and indicate their sleeping times on the actograph. On Monday, an experimenter arrives to install the scent delivery system. This device has been preprogrammed to deliver the distinctive scent 30 minutes before the participant’s regular awakening time. Participants continue to complete the diary and use the actometer in the second week, and starting Monday evening, they also wear the heart rate monitor every night. An experimenter comes by again on Thursday to reset the scent delivery system to go off 4 hours earlier than previously, that is, 4 and a half hours before regular awakening. The experimenter also checks the participant’s experience with the scent device at this time and notes if the participant had any unexpected reactions or has woken up early. Unbeknownst to the participant, if the participant is in the control group, the cartridge containing the scent is also switched for a different cartridge containing a different scent. The participant is instructed to set their alarm 4 hours earlier than normal and perform saliva measures at the new time of awakening and 15, 30, and 45 minutes later. They also use the web-based diary to complete the PANAS, Stress NRS, SVS, and CIS-fatigue directly after the first and third saliva measures. After the final saliva measure, the participants are allowed to return to sleep. They awake at a time of their choosing the following morning and only at that point remove the heart rate monitor. Their only remaining activity that day is a final diary filled in at a time of the participant’s choosing. Later that day, an experimenter picks up the saliva samples and all equipment, including the actometer that the participant can at that point remove, and debriefs the participant about the true aim of the study and their group assignment.

### Data Management

Self-reported data will be entered electronically by participants and checked for completion throughout the study at each experimenter visit. These data will be stored on a secure server that produces daily backups. Saliva samples are stored securely in a freezer at Leiden University Medical Center until study completion. All data are handled in compliance with the Leiden University Data Management Policy (available at request). Personal data are kept separate from the research data, and the file connecting the 2 is secured and only accessible to the study staff. Owing to low risk, external monitoring was deemed unnecessary by the ethical review committee, but a department data manager will monitor the data during the study and check the data package for completeness at study completion. Data will be stored locally for a period of 15 years after study completion and will be uploaded to the Data Archiving and Networked Services database to be accessed at request.

### Statistical Analysis

The data will be checked for outliers and all relevant assumptions. If cortisol values deviate strongly from normality, a log transformation will be performed. An α level of .05 will be used for all analyses, and an effect size (Cohen *d* or partial eta squared, as appropriate) will be calculated for all effects.

Sensitivity analyses will be performed to investigate the effect of including participants who do not show a CAR in week 1 (defined as an increase of at least 2.5 nmol/L above baseline [8]) and the effect of including participants with incomplete data.

A conditioned effect on cortisol values can be defined in 2 ways: by a higher increase in the CAR from baseline (first week) to evocation (second week) for the experimental group (same scent on evocation) than the control group (different scent on evocation), and by a higher increase in the initial cortisol value (first measure of the CAR) from baseline (first week) to evocation (second week) in the experimental group than in the control group. Therefore, 2 separate analyses will be performed. For the first definition, cortisol levels over each day will be transformed into a single score by computing the area under the curve with respect to the first value (area under the curve with respect to increase [7]). We will then perform a linear mixed-model analysis with week (baseline vs evocation) and group (experimental vs control) as fixed factors. For the second definition, we will again perform a linear mixed-model analysis, replacing the area under the curve with respect to increase value with the first cortisol value after awakening. To check for confounding variables, sociodemographic and personality variables will be included as random factors in both the analyses.

Linear mixed models are also used to analyze the secondary outcomes. Heart rate variability measures 30 minutes before awakening and 45 minutes after awakening will be analyzed separately. In both cases, week (baseline vs evocation) and group will again be fixed factors. For the secondary psychological outcomes affect, stress, vitality, and fatigue, the measurement time (0 vs 30 minutes after awakening) is added as an additional fixed factor.

Finally, multivariate regression will be used to explore the possible moderating or mediating role of psychological variables (affect, personality, and perceived stress) on the effects of conditioning.

## Results

Data collection started on June 24, 2019, and we encountered difficulties owing to the COVID-19 pandemic. Data collection was closed on December 24, 2021, with 50 inclusions and 42 participants completing the full 2-week protocol. The results will be published separately at a later date when the data analysis is completed.

## Discussion

The design of this study is intended to classically condition the CAR by repeatedly presenting a scent in the half hour immediately before awakening, immediately before the CAR normally occurs. If the conditioning is successful, participants should respond to the CS with higher cortisol levels even when it appears in the middle of the night, when cortisol levels are normally far below the morning levels. The outcomes of this study could offer more insight into learning during sleep, learning influences on physiological processes, and manipulating the diurnal rhythm. These findings may also aid in the treatment of sleep rhythm and cortisol disorders.

This study uses a novel design that combines the finding that conditioning is possible during sleep [24,25] with the idea that the diurnal rhythm itself could be conditioned [31,32]. While other studies have investigated whether cortisol levels are subject to classical conditioning [19,21-23], all of them have used an external UCS to adjust cortisol levels rather than relying on changes brought upon by the natural diurnal rhythm. Even animal studies that relied on less invasive stimuli kept the animals under artificial lighting conditions that are impossible to replicate in daily human life [31,32]. Should the alternative approach of this study be successful in conditioning the CAR, this could indicate that it is not necessary to rely on medication or highly demanding procedures to influence cortisol levels.

Should the conditioning procedure used in this study be successful in affecting subjective wakefulness measures, then a similar conditioning procedure has potential use in the treatment of sleep disorders. The primary target group would likely be those patients who do not respond to or cannot participate in existing behavioral therapies. Pharmacological therapies currently prescribed for this purpose are not always ideal solutions; many, such as benzodiazepines, do more to deregulate than help the sleep cycle and feature unpleasant side effects and risks of dependence [55].

On the other hand, the conditioning procedure could fail to affect cortisol. This may be due to the novel aspects of the study design. For example, using the stimuli associated with the diurnal rhythm may prove to be less reliable than inducing effects on cortisol through medication; the existing animal studies that relied on this [31,32] may not generalize to humans. Timing the test phase in the middle of the night will hopefully expose the effect because the CAR is not present in the control group, but there are indications that cortisol will still show an increase around an earlier time of awakening as long as the participants are aware that they will awake at the earlier time [33]. There is also the risk that the presence of the scent, the knowledge of having to wake, or the sound of the scent device would cause participants to wake up before the predetermined time. This would cause the scent and CAR to overlap, potentially affecting the conditioning procedure. If the participants do wake up, they could also consciously process the scent. While the debate on the role of consciousness in conditioning is ongoing and beyond the scope of this paper [56], what exactly is learned under these conditions is no doubt different than when asleep. Even if the conditioning procedure still led to an increase in cortisol, the conditioning might not be unconscious or physiological. Therefore, we endeavored to reduce the chances of this occurrence by selecting good sleepers as participants and reducing the noise as much as possible. If the participants do wake up, actigraphy and heart rate variability measures should show this.

Apart from factors specific to this study’s design, a null result may indicate that pharmacological conditioning of cortisol levels in humans may not be feasible in general. The mixed results in existing studies might be the result of underlying confounds or sample fluctuations; several early studies rely on relatively small samples or groups [21,23] or suffer from unexpected baseline differences [19]. We hope that this study will avoid these issues and offer more clarity. The difficulties so far could also indicate that any effect of conditioning on cortisol is small and easily overshadowed by confounds. If the effect is too small or unreliable to be of use in clinical applications, further investigations may not be warranted.

Allowing participants to perform all measures themselves at home will invariably lead to a loss of control over the measurement situation, resulting in overall noisier data and an increased likelihood of missing or inaccurate measurements compared with a laboratory environment. Testing in a laboratory would have also allowed us to perform more continuous measures, such as daily cortisol sampling. However, such a setting would also highly increase participant burden, and sleep in a controlled medical environment has been shown to affect sleep measures in a way that does not occur for at-home measurements [57]. Therefore, we chose to focus on a home setting with fewer measurement points, relying on the reliability of the CAR [9] and stringent participant selection to still provide reliable data. Allowing measures to be taken at home also ensures that the results of the study are generalizable to real-life situations in a way that laboratory measures are not, thus ensuring maximal external validity.

Only healthy young men are included in this study. This selection is performed to prevent possible confounding effects of the menstrual cycle, gender, age, stress, and illness. However, it should be noted that the effects of some of these confounds are small [38] and the findings are often heterogeneous. For example, while some studies find differences in CAR in different phases of the menstrual cycle [58,59], others do not [60,61], and even when there are effects, they are limited to 1 phase differing from all others. Those few studies that cover a large age range show mixed results regarding the effect of age [8,60]. The effect of smoking is similarly equivocal [7,60,62,63]. Because this study uses a novel procedure and the exact size of the expected effect is uncertain, and because testing in the home environment is already going to produce additional noise, precautions have been taken to exclude any additional sources of interference. Thus, we have chosen to control for these factors, even if their effects may be small. However, should the current design produce the expected effect, broadening the type of participants in follow-up studies will greatly help generalizing the results. Extending the design to women may be especially valuable, not only because this is the largest excluded group but also because the CAR tends to be larger in women [7,8,64], which may make it easier to evoke through conditioning.

In conclusion, an innovative study design has been presented to investigate whether the CAR can be classically conditioned during sleep. The design of this study offers a unique first look at the potential of using natural diurnal changes in physiological conditioning. The results of this study will provide clarification on the currently murky topic of the conditioning of cortisol levels and, if positive, can inspire future additive treatment for sleep disorders.

## Abbreviations

CAR: Cortisol Awakening Response
CIS: Checklist Individual Strength
CS: conditioned stimulus
DASS-21: Depression Anxiety Stress Scale-21
HPA axis: Hypothalamus-Pituitary-Adrenal Axis
HRV: heart rate variability
MINI-Plus: Mini-International Neuropsychiatric Interview Plus
NRS: Numerical Rating Scale
PANAS: Positive and Negative Affect Schedule
PSQI: Pittsburgh Sleep Quality Inventory
SVS: Subjective Vitality Scale
UCS: unconditioned stimulus

## Data Availability

Data sharing is not applicable for this paper as no data sets were generated or analyzed during this study.
